# A scoping review on the study of siblings in pediatric pain

**DOI:** 10.1080/24740527.2017.1399053

**Published:** 2017-12-05

**Authors:** Meghan G. Schinkel, Christine T. Chambers, Jill A. Hayden, Abbie Jordan, Justine Dol, Kristen S. Higgins

**Affiliations:** a Departments of Psychology and Neuroscience, Dalhousie University, Halifax, Canada; b Centre for Pediatric Pain Research, IWK Health Centre, Halifax, Canada; c Pediatrics, Dalhousie University, Halifax, Canada; d Community Health and Epidemiology, Dalhousie University, Halifax, Canada; e Department of Psychology, University of Bath, Bath, UK; f Centre for Pain Research, University of Bath, Bath, UK; g Faculty of Health, Dalhousie University, Halifax, Canada

**Keywords:** Children, families, pediatric pain, scoping review, siblings

## Abstract

**Background**: Sibling relationships are longstanding across an individual’s life and are influential in children’s development. The study of siblings in pediatric pain is, although in early stages, a growing field.

**Aims**: This scoping review sought to summarize and map the type of research available examining siblings and pediatric pain to identify gaps and directions for future research.

**Methods**: Studies were identified based on a search of PubMed, CINAHL, PsycInfo, Embase, and Web of Science (up to November 2016). We extracted data about study methods, the sample, outcome assessment, and the influence/relationships investigated.

**Results**: Thirty-five studies were included. Most studies used quantitative methods (*n *= 28), and participants typically included children (i.e., aged 6–12; *n *= 24) and adolescents (i.e., aged 13–18; *n *= 18). The majority of studies examined siblings in the context of chronic and disease-related pain (*n *= 30). Though quantitative studies primarily focused on the genetic influence of pain conditions (*n *= 18), qualitative and mixed-methods studies typically focused on exploring the impact of siblings with and without pain on one another (*n *= 2) and the impact of pain on the broader dyadic relationship/functioning (*n *= 4).

**Conclusions**: Sibling research in pediatric pain has been primarily focused on the biological/physical components of pain, using quantitative approaches. Conducting more studies using qualitative or mixed-methods designs, incorporating multiple assessment measures (e.g., observational, self-report) and multiple perspectives (e.g., siblings, health professionals), may provide an opportunity to gain richer and more comprehensive information regarding the experience of siblings.

## Résumé

**Contexte**: Les relations entre membres d’une même fratrie durent toute la vie et influencent le développement des enfants. Bien qu’elle soit encore à ses débuts, l’étude des fratries dans le domaine de la douleur pédiatrique est un domaine en pleine croissance.

**But**: Cette revue exploratoire avait pour but de cartographier et de résumer le type d’études sur les fratries et la douleur pédiatrique actuellement disponibles, afin de répertorier les lacunes existantes et définir les orientations possibles pour les études à venir.

**Méthodes**: Les études ont été répertoriées par le truchement d’une recherche sur PubMed, CINAHL, PsycInfo, Embase et Web of Science (jusqu’en novembre 2016). Nous en avons tiré des données sur les méthodes, l’échantillon, l’évaluation des résultats et l’influence ou les relations à l’étude.

**Résultats**: Trente-cinq études ont été retenues. La majorité de ces études utilisaient des méthodes quantitatives (*n* = 28) et les participants étaient généralement des enfants (i.e. âgés de 6 à 12 ans; *n* = 24) et des adolescents (i.e. âgés de 14 à 18 ans; *n* = 18). La plupart de ces études portaient sur les frères et soeurs dans le contexte de la douleur chronique liée à une maladie (*n* = 30). Tandis que les études quantitatives portaient généralement sur la dimension génétique de la douleur, (*n* = 18), les études qualitatives et les études mixtes portaient généralement sur l’effet des frères et sœurs avec douleur et sans douleur l’un sur l’autre (*n* = 2) ainsi que sur l’effet de la douleur sur la relation au sein d’une dyade ou le fonctionnement de celle-ci (*n* = 4).

**Conclusions**: La recherche sur les fratries dans le domaine de la douleur pédiatrique met surtout l’accent sur les composantes biologiques/physiques de la douleur, à l’aide d’approches quantitatives. La conduite d’études ayant recours à des devis qualitatifs ou mixtes et intégrant de multiples mesures d’évaluation (i.e. observationnelles, auto-rapportées) et de multiples perspectives (ex. : membres d’une même fratrie, professionnels de la santé) pourrait être une occasion d’obtenir de l’information plus riche et plus exhaustive en ce qui concerne l’expérience des fratries.

A strong body of research has been developed in the field of pediatric pain over the past few decades exploring various aspects of children’s pain.^1^ In particular, research has moved beyond examining only child and adolescent functioning to exploring the wider context of families in pediatric pain. Within chronic pain, models have been developed to elucidate the interplay between family-related variables and children’s experiences of pain and functioning.^2,3^ Studies have also focused on examining parental behavior in the context of children’s acute procedural pain.^4–7^ Laboratory-based studies have added further insight into the influence of familial variables on children’s pain experiences. For example, experimental pain tasks (e.g., the cold pressor task) have been widely utilized to explore family factors in pediatric pain, such as the influence of parent behaviors,^8–10^ parental social modeling,^11^ and family functioning.^12^ Despite a plethora of family-focused research across multiple domains of pediatric pain, siblings have received relatively little attention in the pediatric pain literature. However, siblings are also important family members for children. The majority of children have a sibling,^13^ and research on siblings has outlined the impact that siblings exert on one another with regard to developmental outcomes and psychosocial and behavioral functioning (see Brody^14^ and McHale et al.^15^ for reviews). Further, research on pediatric chronic health issues suggests that a sibling’s experience of illness can adversely influence children’s functioning in several areas, such as psychological symptoms,^16^ quality of life,^17^ and academic participation and performance.^18^

Although limited research exists exploring siblings in pediatric pain, there have been a growing number of both quantitative and qualitative studies focused on the topic. For example, quantitative studies have revealed differences in psychosocial functioning between siblings of healthy children and those with chronic pain conditions, with siblings of pediatric pain patients experiencing poorer functioning, such as anxiety, depression, and social difficulties.^19,20^ Qualitative studies have begun to illustrate the nature of young siblings’ relationships and everyday life when one experiences chronic pain.^21,22^ This work highlights the influence that pain can have on siblings in terms of their personal mental health and relationship with one another and suggests that continued research on the topic has potential to make a valuable contribution to our understanding of the role of families in pediatric pain.

Unlike the more narrow focus of a systematic review, scoping reviews aim to broadly summarize and map research in a given field.^23–25^ They are often conducted when a goal is to determine areas in need of further research.^23^ In contrast to systematic reviews, scoping reviews tend to include studies using a wider array of methods (published or unpublished)^23–25^ and generally focus on describing the literature rather than synthesizing findings to determine effectiveness or the strength/direction of impact.^23,25^ Scoping reviews are considered a useful approach for research areas that are still developing^24,25^ or where the research is varied.^25^ The existing body of research on siblings and pediatric pain is both limited and varied in terms of focus, methodology, and discipline, thus indicating that a scoping review may be an appropriate method for reviewing this area. No known reviews have been conducted on the topic to date. Therefore, the field could benefit from a summary of the work that has been conducted; this may help to identify gaps in the field, stimulate further research, and provide direction moving forward.

In order to provide an overview of the literature to date, the objective of the scoping review was to summarize and map the type of research that has been conducted examining siblings and pediatric pain. Specifically, the review sought to address the question, “What are the characteristics of research studies that have explored the role of siblings in pediatric pain?” This was undertaken with a goal to identify gaps in the literature and directions for future research.

## Methods

The methodological approach was informed by current guidelines for conducting scoping reviews.^23–26^

### Search strategy

A search of the electronic databases PubMed, CINAHL, PsycInfo, Embase, and Web of Science was conducted on November 8, 2016. The search included a combination of terms, formatted for each database, related to siblings (e.g., *sibling, sister, brother*), pain (e.g., *chronic pain, experimental pain, needle*), and children (e.g., *child, pediatrics*). The pain terms included keywords related to chronic pain, experimental pain, and procedural pain and were informed by keywords used in recent reviews in pediatric pain.^27,28^ The child terms represented a validated search strategy for identifying pediatric-focused studies.^29^ Development of the search terms also involved consultation with librarians, who have expertise in conducting searches for reviews, and discussion amongst the co-authors. See Appendix A for the complete search terms used formatted for each database (available as a supplemental document). Additional relevant articles known to the authors based on their knowledge of the literature were also identified for subsequent screening. An additional search was conducted of the electronic databases used in the original systematic search, up to the date of the original search, of the included conference abstracts to ensure that no subsequent published manuscripts based on the abstracts had been missed.

### Eligibility criteria

To be eligible for inclusion, studies had to be pediatric focused, which was defined as including a sample composed of children ages 0–18^1,28,30^ and/or adults reporting on children or adult retrospective studies (i.e., adults reflecting on their experiences as children). Additionally, both siblings and pain or a pain condition had to be of primary interest, as identified in the title and/or abstract. Studies examining siblings in the context of experimental, acute, chronic, or procedural pain were all included. All studies available up until the date of the search that were written in English and reported empirical data or synthesized data using any methodological design, either published or unpublished, were included.

Studies were excluded if they described families broadly with siblings not being a specific focus or included healthy siblings only as a healthy control group. Case studies reporting on more than one sibling who had the same illness (e.g., a case study on a genetic condition), studies referring to pain in an emotional sense, and studies focused on cancer-related pain were excluded. Lastly, articles that were commentaries (i.e., opinion or reaction/reflection-based publications) or letters to the editor were excluded.

### Study selection

**Figure 1.**
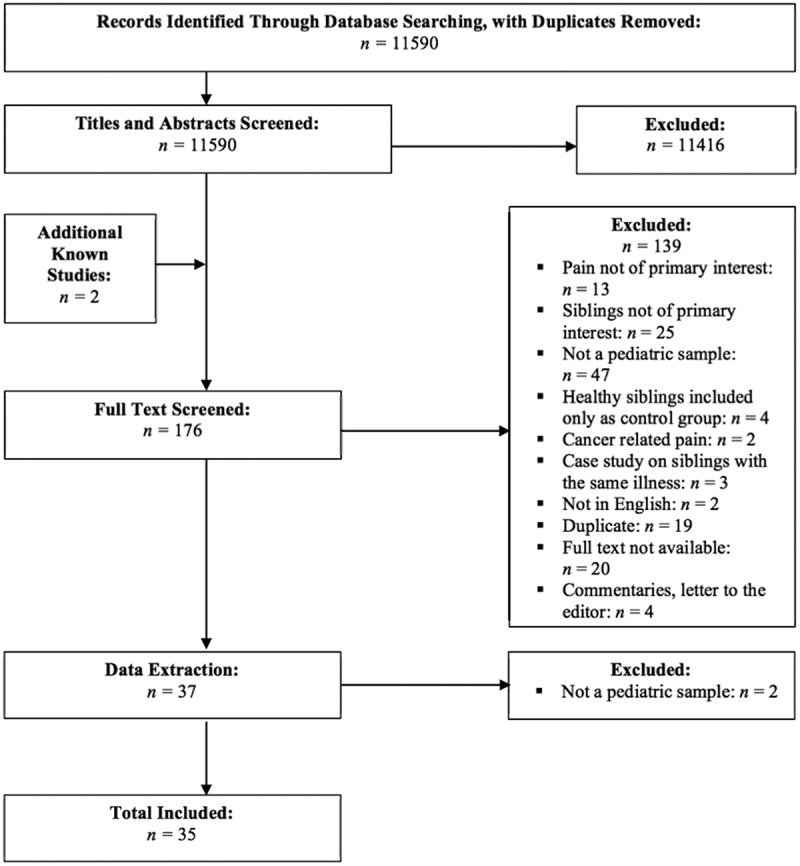
Flowchart outlining the study selection process.

All identified studies were imported into and screened using Covidence,^31^ which is an online screening and data extraction tool designed to help facilitate the review process (see Figure 1 for a flowchart outlining the study selection process). First, MS and JD separately completed a title and abstract screen of all of the identified studies (*n *= 11 590). Discrepancies were resolved by consensus between the two co-authors. For all studies that passed this initial screening stage (*n *= 176), the full text was retrieved and reviewed separately by MS and JD, and discrepancies were again resolved by consensus. Two additional studies were excluded during the data extraction phase due to not reporting on a pediatric sample.

### Data charting

A comprehensive data extraction manual, which provided descriptions of the extraction categories, was developed and underwent several rounds of review by the team of authors prior to charting the data. The full extraction manual is available from the authors. To summarize, we collected

1. descriptive information about the article, including the name of the study, the authors, publication year, geographic location of the study (or, if not listed, the location affiliation of the first author), the discipline affiliation(s) of all authors, and the type of study (published research, dissertation, case study, or conference abstract);
2. information regarding the methodology used (qualitative, quantitative, or review; methodological subcategories were included within each);
3. information about the study sample, including the age of the children (baby/toddler = <2 years old; preschool = 2–5 years old; child = 6–12 years old; adolescent = 13–18 years old; or not specified),^1^ whether adults were included in the study (parents, health professionals, teachers, or adults reporting retrospectively), the type(s) of pain population(s)/context of interest in the study (acute/procedural, chronic/disease related, or experimental; e.g., the cold pressor task; subcategories were included within each), and whether children with a comorbid/other condition of interest (e.g., pain being studied in a group of children with a comorbid, non-pain-related condition) or healthy children (e.g., healthy siblings, healthy children experiencing experimental or acute/procedural pain) were included in the sample;
4. the type(s) of outcomes assessed (demographic variables, quality of life, mental health/psychosocial functioning, adaptive functioning/disability, sibling relationship quality, parent–child relationship quality, parent marital relationship quality, family functioning, pain or somatic symptoms, genetic vulnerability, and juvenile arthritis disease features), how outcomes were measured (questionnaire or survey, observational measures, focus groups, interviews, health records/medical results, pain assessment tools), and who reported on outcomes (parent, healthy/pain free sibling, sibling with pain/condition, health professional) and whether they were reporting on themselves, others (e.g., a parent reporting on their child, a child reporting on their sibling), or having their behavior observed; and, lastly,
5. the influence/relationships investigated in the study in relation to siblings (the impact of the sibling experiencing pain on the healthy/pain free sibling, impact of healthy/pain-free sibling on sibling experiencing pain, bidirectional, impact of/relationship between two siblings with pain/condition on one another, mediating impact of parents or family, the impact of pain on the broader dyadic relationship or functioning, genetic influence). Following initial data extraction, it was determined that “juvenile arthritis disease features” should be an option under the outcomes assessed category, and “genetic influence” (i.e., studies examining siblings within the context of genetic vulnerability for pediatric pain conditions) should be an option under the influence/relationship investigated category. Therefore, these options were subsequently added and relevant studies were recategorized.

The data were charted in Microsoft Excel, which primarily involved indicating the option(s) for each extraction category that best characterized the study. Data from studies could be extracted as falling into more than one option within each category. Data charting was completed for all included studies independently by two co-authors (MS and either JD or KH, who each charted data for half of the studies). Data charting files were compared between reviewers and discrepancies were resolved by consensus.

### Summarizing the results

Microsoft Excel was used to calculate descriptive statistics (e.g., totals, percentages) and to create figures to summarize the data. Descriptive information on all included studies was examined together. The studies were then split based on methodology (quantitative, qualitative, mixed methods), and more detailed results (e.g., participant characteristics, outcomes) were examined separately within each of the methodology categories.

## Results

### Descriptive information

Thirty-five studies were included in the review, representing a total of 21 810 subjects (note: eight studies reported the sample size as the number of participating families). See Appendix B for descriptive information (e.g., publication year, discipline) of the included studies (available as a supplemental document). The majority of included studies were published research studies (*n *= 21), with the remainder including conference abstracts (*n *= 12) or dissertations (*n *= 2; Table 1). No subsequent published manuscripts based on the included conference abstracts were identified in the search. Most of the research papers (or studies) were classified as quantitative (*n *= 28), although some qualitative studies have been conducted (*n *= 5). Additionally, two studies were mixed methods, utilizing both quantitative and qualitative methodology. We did not identify any reviews conducted in the field as of the date of the search (Table 1).

**Table 1.** Type of study and methodology used across the included studies.

Study	Study type			Methodology	
	Published research study	Dissertation	Conference abstract	Qualitative	Quantitative
Guite et al.^19^	X				X
Engstrom^20^	X				X
Gorodzinksy et al.^21^	X			X	
Britton and Moore^22^	X			X	
Badiee et al.^32^	X				X
Barton et al.^33^			X		X
Campbell-Yeo et al.^34^	X				X
Campbell et al.^35^			X		X
Campbell-Yeo et al.^36^	X				X
Champion et al.^37^	X				X
Champion et al.^38^			X		X
Chan et al.^39^			X		X
El-Metwally et al.^40^	X				X
Field et al.^41^	X				X
Filocamo et al.^42^			X		X
Flynn et al.^43^			X		X
Gordon^44^		X		X	
Gunalan et al.^45^			X		X
Kofman et al.^46^			X		X
Lee et al.^47^			X		X
McOmber and Shulman^48^			X		X
Mikkelsson et al.^49^	X				X
Miller et al.^50^	X				X
Moroldo et al.^51^	X				X
Moroldo et al.^52^	X				X
Moscato et al.^53^			X	X	
Prahalad et al.^54^	X				X
Saila et al.^55^	X				X
Scherder et al.^56^	X				X
Stahl et al.^57^	X				X
Svensson et al.^58^	X				X
Valkenburg et al.^59^	X			X	X
Wong et al.^60^			X		X
Wutzke^61^		X		X	X
Akobeng et al.^62^	X			X	

### Quantitative studies

#### Methods

Experimental/quasi-experimental (*n *= 10) and nonexperimental methods (*n *= 12) were used in a similar number of studies, with fewer studies using a cross-sectional design (*n *= 6). Almost all studies were classified as including a descriptive component (i.e., reporting descriptive findings; *n *= 23). Although longitudinal and measurement (e.g., questionnaire development) were included as options, no studies were extracted as falling into these categories.

#### Sample

**Figure 2.**
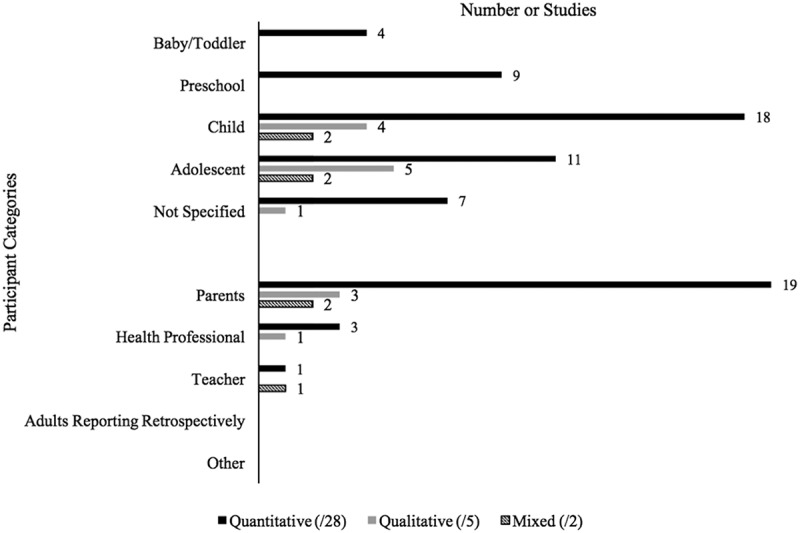
Participant characteristics of included studies by methodology type across age of pediatric sample and adult involvement.

Most studies included participants in the child (*n *= 18) or adolescent (*n *= 11) age categories, with fewer studies including preschool-aged children (*n *= 9) or babies/toddlers (*n *= 4; Figure 2). It should be noted that seven studies did not specify the age of their pediatric sample. Adults also often participated in the quantitative studies. Most studies included parents (*n *= 19), with three studies also including health professionals^51,52,58^ and one study including teachers.^56^ None of the examined studies included adults reporting retrospectively on their childhood (Figure 2).

With regard to the type of pain examined, almost all studies were focused on chronic or disease-related pain (*n *= 24), with the most common pain sample being juvenile arthritis/rheumatic diseases (*n* = 6). Four studies were focused on acute/procedural pain,^32,34,36,56^ and only one examined pain in the context of an experimental task^56^ (Table 2). In addition, three studies included a sample of children with co-morbid or other conditions of interest.^41,56,^^60^ Most studies (*n *= 21) included healthy children in their sample.

**Table 2.** Type of pain examined across included studies by methodology type.

	Number of studies		
Pain type	Quantitative (/28)	Qualitative (/5)	Mixed (/2)
Acute/procedural	4	0	0
Needle/immunization	0		
Blood draw	4		
Postoperative	0		
Other	1		
Chronic/disease related	24	5	1
Chronic pain	0	0	0
Irritable bowel disease/syndrome	1	0	0
Inflammatory bowel disease	1	2	0
Migraine/headache	2	2	0
Juvenile arthritis/rheumatic diseases	6	1	1
Abdominal pain	3	1	0
Back pain	3	0	0
Musculoskeletal	2	1	0
Growing pains	4	0	0
Sickle cell disease	2	0	0
Other	1	1	0
Experimental	1	0	1
Cold pressor	0		0
Quantitative sensory testing	0		1
Water Load Task	0		0
Other	1		0

#### Outcomes

**Figure 3.**
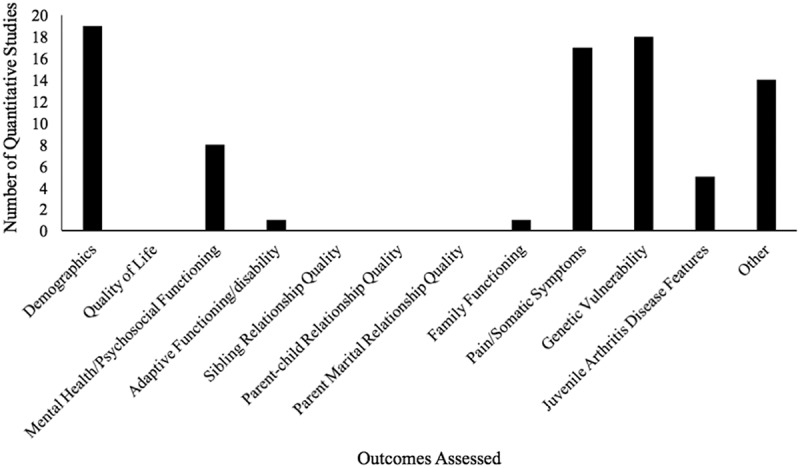
Number of quantitative studies assessing extracted outcomes.

Most of the quantitative studies examined demographic variables (e.g., socioeconomic status; *n *= 19). Following demographics, the most frequently assessed outcomes were genetic vulnerability (*n = *18) and pain or somatic symptoms (e.g., pain severity, condition-related symptoms; *n = *17). Mental health/psychosocial functioning was also of interest in a number of studies (*n = *8; Figure 3). Many studies assessed outcomes that did not fall into one of the extraction categories, and these were varied in nature such that they could not be meaningfully categorized (e.g., malaria history,^35^ co-sleeping with a parent, sibling, or pet during first year of life^50^).

#### Outcome assessment

**Figure 4.**
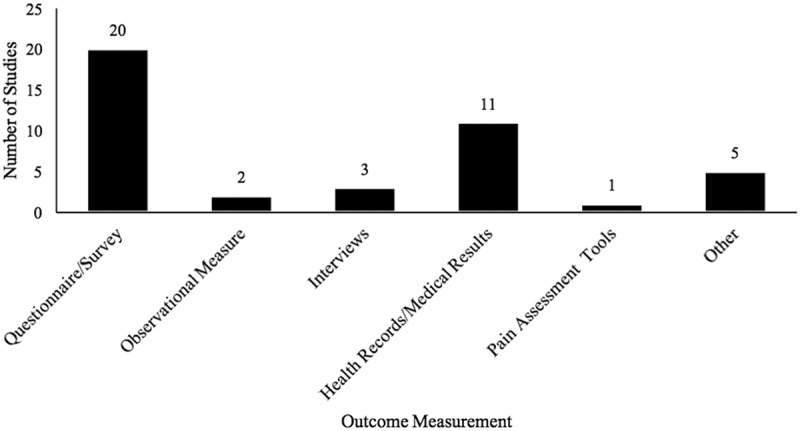
Type of outcome measurement used across quantitative studies. Note: No identified studies met inclusion criteria for the “focus group” extraction option.

Most studies relied on questionnaires or surveys to assess outcomes (*n* = 20), followed by health records or medical results (*n* = 11). Only two studies utilized observational measures^32,34^ and only one study used pain assessment tools^56^ (Figure 4). Half of the studies (*n* = 14) used parent report to assess outcomes. Within these studies, 93% of parents reported on others and 43% reported outcomes on themselves. Healthy/pain free siblings (*n* = 8) and siblings with pain (*n* = 11) reported on outcomes in less than half of the studies. Within both categories, most children reported on themselves. Of the three studies that used health professionals to report on outcomes, all reported on others (Table 3).

**Table 3.** Sources of information for outcome assessment across quantitative studies.

Informant	Number of studies	% Within category
Parent	14	
Self-report		43
Reporting on others (e.g., children)		93
Behavior observed		0
Healthy/pain-free sibling	8	
Self-report		100
Reporting on others (e.g., sibling w/pain)		0
Behavior observed		0
Sibling(s) with pain/condition	11	
Self-report		82
Reporting on others (e.g., healthy sibling)		0
Behavior observed		18
Health professional	3	
Self-report		0
Reporting on others (e.g., children)		100
Behavior observed		0
Other	1

#### Influence/relationships investigated

**Figure 5.**
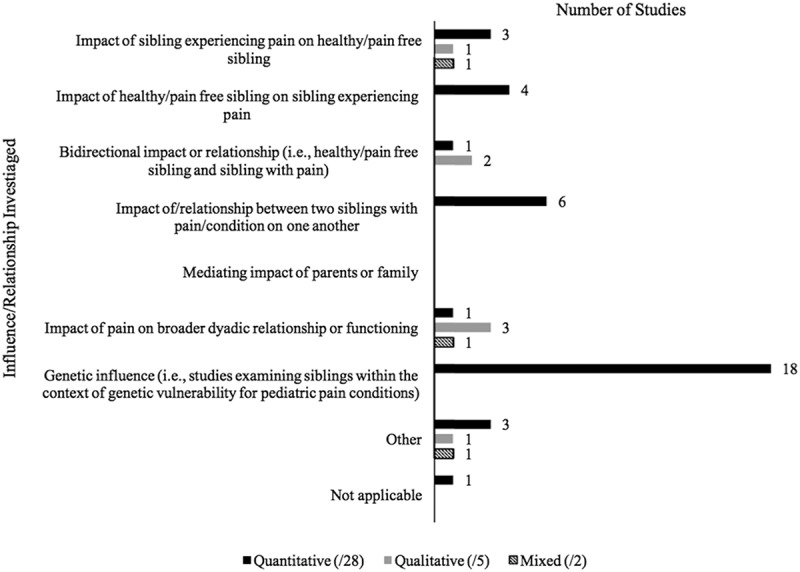
The influence/relationship investigated regarding siblings in pediatric pain by methodology type.

The majority of quantitative studies were focused on siblings in the context of a genetic influence/vulnerability for a pediatric pain condition (*n* = 18). This was followed by studies examining the impact of/relationship between two siblings with pain/a condition on one another (*n* = 6; Figure 5). No studies examined the mediating impact of parents or family (e.g., examining how parent mental health mediates the impact of child chronic pain on a healthy sibling).

### Qualitative studies

#### Methods

Interviews (*n* = 3),^21,22,44^ a qualitative questionnaire (*n *= 1),^22^ and focus groups (*n *= 1)^62^ were used to obtain data in the qualitative studies, with three studies also using “other” means (e.g., drawings^53^). To analyze the qualitative data, one study reported using inductive content analysis,^44^ one grounded theory,^22^ and one the Delphi coding procedure.^21^ Two studies reported using “other” qualitative analytic approaches (e.g., describing qualitative findings from a projective test^53^).

#### Sample

Aligning with the quantitative studies, most qualitative studies included participants within the child (*n* = 4) and adolescent (*n *= 5) age ranges. Parents were also included in three of the qualitative studies,^22,44,62^ and health professionals were included in one study^53^ (Figure 2). All of the qualitative studies were focused on chronic/disease-related pain, with the specific disease of interest varying across studies (Table 2). No studies included a sample of children with co-morbid or other conditions of interest, but all studies had healthy children included in the sample.

#### Influence/relationships investigated

Three of the qualitative studies were focused on the impact of pain on the broader dyadic relationship or functioning,^21,44,53^ with two studies also focused on the bidirectional impact of siblings with pain and healthy/pain-free siblings on one another.^21,44^ One study was focused solely on the impact of the sibling with pain on the healthy/pain free sibling,^62^ and one study was classified as “other” and was focused on the general experiences of families of children with juvenile idiopathic arthritis^22^ (Figure 5).

### Mixed-methods studies

#### Methods

Within the two mixed-methods studies, one used an experimental/quasi-experimental design^59^ and the other a nonexperimental design,^61^ with both including a descriptive component. To analyze the qualitative data, one study reported using thematic analysis^61^ and the other study did not clearly specify their approach but reported using qualitative questions to obtain data.^59^

#### Sample

Both studies included participants in the child and adolescent age ranges (*n *= 2). Parents were included in both studies, with one study also including teachers^61^ (Figure 2). One of the studies was focused on chronic pain,^61^ whereas the other examined pain in the context of an experimental task^59^ (Table 2). One of the studies included a sample of children with a co-morbid or other condition of interest^59^ and both included healthy children in the sample.

#### Outcomes

Demographics were assessed in both studies, with the following outcomes additionally being assessed in either one of the two studies: mental health/psychosocial functioning,^61^ adaptive functioning/disability,^59^ sibling relationship quality,^61^ parent–child relationship quality,^61^ and pain/somatic symptoms.^59^ Both studies also assessed outcomes that fell in the “other” category (e.g., reaction time of nondominant hand,^59^ general experience of having a sibling with juvenile rheumatoid arthritis^61^).

#### Outcome assessment

Outcomes were measured using questionnaires in both studies, with one study additionally using observational measures, pain assessment tools, and “other” measurement tools^59^ and the other study additionally using interviews.^61^ Parents (reporting on others) and healthy/pain-free siblings (self-report) provided information in both studies, with a sibling with pain/a pain condition additionally reporting on him- or herself in one of the studies.^59^ A teacher also provided information in one of the studies.^61^

#### Influence/relationships investigated

One of the studies focused on the impact of the sibling with pain on the healthy/pain-free sibling as well as the impact of pain on the broader dyadic relationship or functioning.^61^ The impact investigated in the second study was extracted as falling into the “other” option and was focused on pain in children with Down syndrome and their siblings^59^ (Figure 5).

## Discussion

This scoping review sought to summarize and map the research conducted to date on siblings and pediatric pain, with an aim to identify gaps in the literature and directions for future research. Limited research on the topic was identified. Only 60% of the 35 included studies were published research studies, suggesting that the field is still developing and that more research is needed. Regarding methodology, most identified studies were quantitative, using either experimental/quasi-experimental or nonexperimental designs. A small group of qualitative studies has also been conducted, and they varied in terms of their approach to obtaining data and analyzing findings. Only two mixed-methods studies were identified. Therefore, the field has taken a primarily quantitative approach to understanding siblings and pediatric pain, with less focus thus far on obtaining qualitative information regarding participants’ perspectives and experiences or using complementary mixed-methods approaches.

The findings pertaining to the sample characteristics were generally consistent across methodology type. Concurrent with the broader pediatric pain literature,^1^ participants were most often in the child and adolescent age groups. However, a notable number of studies included preschool-aged children or babies/toddlers (combined *n *= 13). Therefore, research examining siblings in pediatric pain is generally well distributed across the pediatric age span. Parents were typically included in the studies examined, suggesting that information pertaining to, or reported by, parents has been valued in the field thus far. Chronic and disease-related pain were the predominant context in which research has examined siblings in pediatric pain, with less attention paid to the potential role of siblings in acute pain experiences. Further, most studies included healthy children in their sample, likely reflecting an inclusion of healthy siblings of chronic pain patients.

Within the quantitative studies, the most commonly assessed outcomes were genetic vulnerability and pain or somatic symptoms, suggesting a focus thus far on the biological or physical components of pediatric pain. Mental health and/or psychosocial functioning were also assessed in several studies, indicating that research on siblings has also examined, albeit to a lesser extent, psychological factors related to pain. Questionnaires and surveys were the predominant means of assessing outcomes for quantitative studies. Parents were a primary source of information, with parents reporting on outcomes in half of the studies. Children (i.e., healthy siblings or siblings with pain) also provided information, although less frequently (less than half of the included studies). Therefore, research findings have been primarily based on parent questionnaire report, with less focus on obtaining children’s perspectives or garnering information from behavioral observation.

Regarding the influence/relationships investigated, the findings for the quantitative studies mirrored that of the outcomes assessed; most studies were interested in siblings in the context of a genetic influence/vulnerability for a pain condition. However, a difference was noted across the methodology types. Unlike quantitative studies, qualitative and mixed-methods studies more often focused on the impact of children on their siblings and the impact of pain on the broader dyadic relationship or functioning. Therefore, much of our understanding of siblings’ functioning and broader experiences come from a qualitative perspective, with limited supporting quantitative data on these topics.

### Identified gaps and directions for future research

As evidenced by the limited numbers of studies in specific areas, gaps were noted regarding the methodology, samples, outcome assessment, and the outcomes and influence/relationships investigated, suggesting some relevant directions for continued research.

First, in terms of methodology, limited qualitative studies exist focusing on siblings and pediatric pain. Given that it is a relatively new field, conducting more qualitative research with patients, families, and clinicians may serve as a means for identifying predominant issues and concerns from the perspectives of those who are most impacted. Further, qualitative methodology typically involves encouraging participants to provide detailed, nondirected responses to open-ended questions on specific topics. Therefore, qualitative studies may offer more in-depth and detailed information regarding specific aspects of individuals’ personal experiences than that which may be obtained through quantitative methods alone (e.g., questionnaires). This richer understanding could also contribute to the development of theoretical models regarding how siblings may impact, and be impacted by, children’s pain experiences. Topics of importance identified through qualitative studies could then be further explored using qualitative, quantitative, or mixed-methods designs. Consistent with limitations identified in sibling research^63,64^ and family research in pediatric pain,^2,3^ no longitudinal studies were identified in the current review. Longitudinal designs could usefully be conducted to answer potentially important research questions, such as the impact of pediatric chronic pain on siblings across developmental stages or the impact of viewing a sibling undergo a painful procedure on a healthy child’s subsequent experience. Further, no measurement studies were identifed. In order for the field to grow, more validated self-report and observational measures pertaining to siblings and pediatric pain will need to be developed.

Second, regarding the samples used, almost all studies, regardless of methodology, concentrated on siblings in the context of chronic or disease-related pain. This is certainly a valuable area for continued research. However, attention should also be given to siblings in the context of acute procedural pain (e.g., surgery, needles) or everyday pains (e.g., illness, injuries). Approximately 98% of parents report bringing siblings to their child’s medical appointments, with 85% specifically reporting bringing siblings to needle procedures^65^ supporting the relevance of exploring the influence of siblings in these acute pain settings. Quantitative and qualitative designs could be used to answer any number of relevant research questions, such as siblings’ impact on children’s procedural pain or distress or children’s responses to their siblings’ common pains at home. Only two studies were identified that examined experimental pain. Experimental pain tasks offer a more standardized approach to studying pediatric pain^66^ and thus have the potential to provide valuable insight into sibling factors relevant to chronic or acute pain. For example, using a standardized experimental pain task, researchers could compare child responses to a pain stimulus with or without a sibling present. Differences in child responses could then be attributed to the presence of the sibling with a greater degree of confidence than could be afforded in a more unpredictable clinical context. Guidelines exist that could be used to assist researchers in identifying the most appropriate pain task for the research question at hand (see Birnie et al.^66^).

Third, regarding outcome assessment, most quantitative studies on siblings and pediatric pain used questionnaires to assess outcomes. A frequent dependence on questionnaires and need to begin to use other forms of outcome assessment have been noted both in sibling research^64,65^ and in research on families in pediatric pain.^2,3^ Research on siblings and pain could begin to use other assessment measures, such as observational measures or pain assessment tools, to provide richer and more comprehensive information. Further, very few studies included health professionals. Incorporating health professionals is likely valuable because they could offer a unique perspective on the outcomes of interest. They may also have insight into other important topics for research on siblings, relevant to chronic or acute pain, based on their experiences working with families.

The findings regarding the source of information for outcome assessment among the quantitative studies suggest that most studies did not use a multi-informant approach. As noted above, half of the studies used parent report, and less than half of the studies included children themselves (i.e., healthy siblings or siblings with pain) to provide information on outcomes. This finding has both empirical and theoretical implications. As recommended for family research in pediatric pain,^2^ future research on siblings should incorporate the perspectives of multiple family members when possible, including all relevant caregivers and siblings, to obtain a complete picture of the issue of interest. This is important because studies on siblings of children with health issues, including pain, have noted discrepancies between reports within family members.^17,19,21^ From a theoretical perspective, the need for theory-guided studies on siblings and pediatric pain has been noted.^67^ Research in this area would be wise to capitalize on the well-developed models that already exist on families and pediatric pain (see Palermo and Chambers^2^ and Palermo et al.^3^). However, these models view relations between family and child variables relevant to pediatric pain as being bidirectional.^2,3^ Therefore, a multi-informant approach to the study of siblings is warranted to build on our existing theoretical understanding of families and pediatric pain.

Lastly, the outcomes and influence/relationships investigated in the included studies suggest a strong focus thus far, particularly among quantitative studies, on genetic factors related to pediatric pain conditions. Pediatric health issues more broadly can influence the functioning and experiences of healthy siblings across a number of domains (e.g., psychological and social functioning, daily life, academics),^16–18,63,68^ pointing to the relevance of further exploring these variables in siblings of chronic pain patients. An examination of the results of the quantitative^19,20^ and mixed-methods^61^ studies included in the current review that examined the impact of chronic pain on healthy siblings’ psychosocial functioning revealed some convergent findings and point to a generally negative influence. Specifically, as noted in the Introduction, two quantitative studies similarly found that siblings of children with chronic pain conditions have significantly more social/peer difficulties and greater anxiety and depression compared to control groups of siblings of healthy children.^19,20^ Further, although no control group was included, a mixed-methods dissertation found that seven of the ten healthy siblings of children with juvenile rheumatoid arthritis included in the study were identified (based on self-, parent, and/or teacher report) as having significant difficulties on a measure of psychosocial functioning (e.g., regarding internalizing behaviors, externalizing behaviors).^61^ Thus, continued research is needed that focuses on other potentially important factors related to siblings and pediatric pain, such as psychosocial and adaptive functioning, quality of life, family functioning and relationships, or social determinants of health. Research focused on a broader array of outcomes among both healthy siblings and those with chronic pain conditions will provide a more comprehensive account of the influence that siblings may have in both chronic and acute pain contexts. Further, although 18 years was used as the upper age limit for studies included in the current review, examining sibling relationships in the context of pediatric pain during older adolescents/early adulthood would be a valuable direction for future research, because sibling impacts may differ as older adolescents leave the family home.

### Limitations

There are several limitations to the current scoping review that should be noted. Although the search strategy was developed to capture all potentially relevant studies, it is possible that some relevant studies were missed. Further, the scoping review did not differentiate between studies based on their sample size or type (i.e., published research, abstract, dissertation), and the quality of the included research studies or the potential strength of their findings was not assessed. This may be particularly important to consider given the high number of included studies that were not published research (e.g., conference abstracts with no identified corresponding peer-reviewed manuscript) and therefore may not have been exposed to the same level of scrutiny as standard peer review. Further, stakeholder consultation has been suggested as a step that could be undertaken when conducting a scoping review.^23,24^ Given that the results of the review confirmed that the field is still in early stages of development, it was decided that formally conducting a stakeholder consultation would not add significant value. However, the review findings and potential interpretations were formally discussed among the co-authors, who include individuals engaged in family research in pediatric pain. Engaging stakeholders, including clinicians and families, in study design and implementation of research focused on siblings and pediatric pain will be valuable as the field progresses.

## Conclusion

The findings of this scoping review suggest that research on siblings in pediatric pain is a growing field. Although some areas emerged as being further developed than others, such as research using quantitative methods and studies focused on genetics and chronic/disease-related pain, continued research is needed across many domains. Theoretical models on families and pediatric pain (see Palermo and Chambers^2^ and Palermo et al.^3^) could be applied to research on siblings to provide both a theoretical foundation, as well as ideas for relevant research questions. As the field develops, the role of siblings should be more explicitly incorporated into these family models. Although the best research design will be informed by the question of interest, a mixed-methods approach using multiple informants will likely yield the most meaningful information. Validated tools relating to siblings and pediatric pain, including both observational and self-report measures, need to be developed to adequately address relevant research questions. Once sufficient research exists examining specific research questions or outcomes pertaining to siblings, conducting a systematic review and meta-analysis will be an important next step in developing an evidence base. For example, genetic factors pertaining to pediatric pain conditions or psychosocial outcomes of siblings of children with chronic pain would be meaningful topics for systematic reviews once the literature is more developed. It is hoped that the findings of this review can be used as a guide for researchers interested in furthering the understanding of siblings in pediatric pain.

## Appendix A: Complete search terms

**Table:**

Sibling terms	Pain terms	Child terms^29^
Siblings Sibling Twins Twin Sister Brother Multiple birth offspring	Chronic pain terms^27^: pain* fibromyalgia irritable bowel syndrome arthrit* osteoarthrit* headache* migraine* neuralgi* neuropath* complex regional pain syndrome chronic pain arthritis osteoarthritis headache migraine neuralgia peripheral nervous system diseases Experimental pain terms^28^: experimental pain cold pressor quantitative sensory test water load heat pain thermal pain pressure pain exercise task Procedural pain terms: needle surgery puncture operation blood draw	Infan* newborn* new-born* perinat* neonat* baby baby* babies toddler* minors minors* boy boys boyfriend boyhood girl* kid kids child child* children* schoolchild* schoolchild school child adolescen* juvenil* youth* teen* under*age* pubescen* pediatrics pediatric* paediatric* peadiatric* school school* prematur* preterm*
Pub Med search format: “siblings”[MeSH] OR sibling*[tw] OR “Twins”[MeSH] OR Twin*[tw] OR sister*[tw] OR brother*[tw] OR “Multiple Birth Offspring”[MeSH]	Pub Med search format: Pain*[tiab] OR Fibromyalgia[tiab] OR Irritable bowel syndrome[tiab] OR Arthrit*[tiab] OR Osteoarthrit*[tiab] OR Headache*[tiab] OR Migraine*[tiab] OR Neuralgi*[tiab] OR Neuropath*[tiab] OR Complex regional pain syndrome[tiab] OR Pain[MeSH:NoExp] OR Chronic Pain[MeSH] OR Fibromyalgia[MeSH:NoExp] OR Irritable Bowel Syndrome[MeSH:NoExp] OR Arthritis[MeSH:NoExp] OR Osteoarthritis[MeSH:NoExp] OR Headache[MeSH:NoExp] OR Migraine[MeSH:NoExp] OR Neuralgia[MeSH:NoExp] OR Peripheral Nervous System Diseases[MeSH:NoExp] OR Complex Regional Pain Syndromes[MeSH:NoExp] OR Needle[tiab] OR Surgery[tiab] OR Puncture[tiab] OR Operation[tiab] OR Blood draw[tiab] OR experimental pain[tiab] OR cold pressor[tiab] OR quantitative sensory test[tiab] OR water load[tiab] OR heat pain[tiab] OR thermal pain[tiab] OR pressure pain[tiab] OR exercise task[tiab]	Pub Med search format: Infan* OR newborn* OR new-born* OR perinat* OR neonat* OR baby OR baby* OR babies OR toddler* OR minors OR minors* OR boy OR boys OR boyfriend OR boyhood OR girl* OR kid OR kids OR child OR child* OR children* OR schoolchild* OR schoolchild OR school child[tiab] OR school child*[tiab] OR adolescen* OR juvenil* OR youth* OR teen* OR under*age* OR pubescen* OR pediatrics[MeSH] OR pediatric* OR paediatric* OR peadiatric* OR school[tiab] OR school*[tiab] OR prematur* OR preterm*
Cinahl search format: MH “siblings” OR TX sibling* OR MH “Twins” OR TX Twin* OR TX sister* OR TX brother* OR MH “Multiple Birth Offspring”	Cinahl search format: TI Pain* OR AB Pain* OR TI Fibromyalgia OR AB Fibromyalgia OR TI Irritable bowel syndrome OR AB Irritable bowel syndrome OR TI Arthrit* OR AB Arthrit* OR TI Osteoarthrit* OR AB Osteoarthrit* OR TI Headache* OR AB Headache* OR TI Migraine* OR AB Migraine* OR TI Neuralgi* OR AB Neuralgi* OR TI Neuropath* OR AB Neuropath* OR TI Complex regional pain syndrome OR AB Complex regional pain syndrome OR MH Pain OR MH Chronic Pain OR MH Fibromyalgia OR MH Irritable Bowel Syndrome OR MH Arthritis OR MH Osteoarthritis OR MH Headache OR MH Migraine OR MH Neuralgia OR MH Peripheral Nervous System Diseases OR MH Complex Regional Pain Syndromes OR TI Needle OR AB Needle OR TI Surgery OR AB Surgery OR TI Puncture OR AB Puncture OR TI Operation OR AB Operation OR TI Blood draw OR AB Blood draw OR TI experimental pain OR AB experimental pain OR TI cold pressor OR AB cold pressor OR TI quantitative sensory test OR AB quantitative sensory test OR TI water load OR AB water load OR TI heat pain OR AB heat pain OR TI thermal pain OR AB thermal pain OR TI pressure pain OR AB pressure pain OR TI exercise task OR AB exercise task	Cinahl search format: Infan* OR newborn* OR new-born* OR perinat* OR neonat* OR baby OR baby* OR babies OR toddler* OR minors OR minors* OR boy OR boys OR boyfriend OR boyhood OR girl* OR kid OR kids OR child OR child* OR children* OR schoolchild* OR schoolchild OR TI school child OR AB school child OR TI school child* OR AB school child* OR adolescen* OR juvenil* OR youth* OR teen* OR under*age* OR pubescen* OR MH pediatrics OR pediatric* OR paediatric* OR peadiatric* OR TI school OR AB school OR TI school* OR AB school* OR prematur* OR preterm*
PsycINFO search format: DE “siblings” OR TX sibling* OR DE “Twins” OR TX Twin* OR TX sister* OR TX brother* OR DE “Multiple Birth Offspring”	PsycINFO search format: TI Pain* OR AB Pain* OR TI Fibromyalgia OR AB Fibromyalgia OR TI Irritable bowel syndrome OR AB Irritable bowel syndrome OR TI Arthrit* OR AB Arthrit* OR TI Osteoarthrit* OR AB Osteoarthrit* OR TI Headache* OR AB Headache* OR TI Migraine* OR AB Migraine* OR TI Neuralgi* OR AB Neuralgi* OR TI Neuropath* OR AB Neuropath* OR TI Complex regional pain syndrome OR AB Complex regional pain syndrome OR DE Pain OR DE Chronic Pain OR DE Fibromyalgia OR DE Irritable Bowel Syndrome OR DE Arthritis OR DE Osteoarthritis OR DE Headache OR DE Migraine OR DE Neuralgia OR DE Peripheral Nervous System Diseases OR DE Complex Regional Pain Syndromes OR TI Needle OR AB Needle OR TI Surgery OR AB Surgery OR TI Puncture OR AB Puncture OR TI Operation OR AB Operation OR TI Blood draw OR AB Blood draw OR TI experimental pain OR AB experimental pain OR TI cold pressor OR AB cold pressor OR TI quantitative sensory test OR AB quantitative sensory test OR TI water load OR AB water load OR TI heat pain OR AB heat pain OR TI thermal pain OR AB thermal pain OR TI pressure pain OR AB pressure pain OR TI exercise task OR AB exercise task	PsycINFO search format: Infan* OR newborn* OR new-born* OR perinat* OR neonat* OR baby OR baby* OR babies OR toddler* OR minors OR minors* OR boy OR boys OR boyfriend OR boyhood OR girl* OR kid OR kids OR child OR child* OR children* OR schoolchild* OR schoolchild OR TI school child OR AB school child OR TI school child* OR AB school child* OR adolescen* OR juvenil* OR youth* OR teen* OR under*age* OR pubescen* OR DE pediatrics OR pediatric* OR paediatric* OR peadiatric* OR TI school OR AB school OR TI school* OR AB school* OR prematur* OR preterm*
Embase search format: “siblings”/exp OR sibling* OR “Twins”/exp OR Twin* OR sister* OR brother* OR “Multiple Birth Offspring”/exp	Embase search format: Pain*:ti,ab OR Fibromyalgia:ti,ab OR “Irritable bowel syndrome”:ti,ab OR Arthrit*:ti,ab OR Osteoarthrit*:ti,ab OR Headache*:ti,ab OR Migraine*:ti,ab OR Neuralgi*:ti,ab OR Neuropath*:ti,ab OR “Complex regional pain syndrome”:ti,ab OR ‘Pain’/exp OR ‘Chronic Pain’/exp OR ‘Fibromyalgia’/exp OR ‘Irritable Bowel Syndrome’/exp OR ‘Arthritis’/exp OR ‘Osteoarthritis’/exp OR ‘Headache’/exp OR ‘Migraine’/exp OR ‘Neuralgia’/exp OR ‘Peripheral Nervous System Diseases’/exp OR ‘Complex Regional Pain Syndromes’/exp OR Needle:ti,ab OR Surgery:ti,ab OR Puncture:ti,ab OR Operation:ti,ab OR “Blood draw”:ti,ab OR “experimental pain”:ti,ab OR “cold pressor”:ti,ab OR “quantitative sensory test”:ti,ab OR “water load”:ti,ab OR “heat pain”:ti,ab OR “thermal pain”:ti,ab OR “pressure pain”:ti,ab OR “exercise task”:ti,ab	Embase search format: Infan* OR newborn* OR new-born* OR perinat* OR neonat* OR baby OR baby* OR babies OR toddler* OR minors OR minors* OR boy OR boys OR boyfriend OR boyhood OR girl* OR kid OR kids OR child OR child* OR children* OR schoolchild* OR schoolchild OR “school child”:ti,ab OR “school child*”:ti,ab OR adolescen* OR juvenil* OR youth* OR teen* OR under* NEXT/1 age* OR pubescen* OR ‘pediatrics’/exp OR pediatric* OR paediatric* OR peadiatric* OR school:ti,ab OR school*:ti,ab OR prematur* OR preterm*
Web of Science search format: “siblings” OR sibling* OR “Twins” OR Twin* OR sister* OR brother* OR “Multiple Birth Offspring”	Web of Science search format: Pain* OR Fibromyalgia OR Irritable bowel syndrome OR Arthrit* OR Osteoarthrit* OR Headache* OR Migraine* OR Neuralgi* OR Neuropath* OR Complex regional pain syndrome OR Pain OR Chronic Pain OR Fibromyalgia OR Irritable Bowel Syndrome OR Arthritis OR Osteoarthritis OR Headache OR Migraine OR Neuralgia OR Peripheral Nervous System Diseases OR Complex Regional Pain Syndromes OR Needle OR Surgery OR Puncture OR Operation OR Blood draw OR experimental pain OR cold pressor OR quantitative sensory test OR water load OR heat pain OR thermal pain OR pressure pain OR exercise task	Web of Science search format: Infan* OR newborn* OR new-born* OR perinat* OR neonat* OR baby OR baby* OR babies OR toddler* OR minors OR minors* OR boy OR boys OR boyfriend OR boyhood OR girl* OR kid OR kids OR child OR child* OR children* OR schoolchild* OR schoolchild OR school child OR school child* OR adolescen* OR juvenil* OR youth* OR teen* OR under*age* OR pubescen* OR pediatrics OR pediatric* OR paediatric* OR peadiatric* OR school OR school* OR prematur* OR preterm*

## Appendix B: Table representing descriptive information for included studies

**Table:**

	Number of studies
Publication year	
1992	1
1997	1
1999	3
2000	1
2001	2
2002	1
2004	1
2007	1
2008	3
2009	3
2010	1
2011	2
2012	4
2013	7
2014	2
2015	2
Country	
Australia	10
Canada	5
Finland	4
Ghana	1
Iran	1
Italy	1
Sweden	2
The Netherlands	2
United Kingdom	2
United States	8
Unknown	2
Discipline^a^	
Medicine	19
Psychiatry	5
Psychology	7
Nursing	2
Physiotherapy	1
Other	14
Not listed	10

^a^For “discipline”, no studies were extracted as “genetics” or “occupational therapy.”
